# *catena*-Poly[[lead(II)-μ-(2-oxidobenz­aldehyde isonicotinoylhydrazonato)] methanol monosolvate]

**DOI:** 10.1107/S1600536811043078

**Published:** 2011-10-22

**Authors:** Gholam Hossein Shahverdizadeh, Seik Weng Ng, Edward R. T. Tiekink, Babak Mirtamizdoust

**Affiliations:** a Department of Chemistry, Faculty of Science, Tabriz Branch, Islamic Azad University, PO Box 1655, Tabriz, Iran; b Department of Chemistry, University of Malaya, 50603 Kuala Lumpur, Malaysia; c Chemistry Department, Faculty of Science, King Abdulaziz University, PO Box 80203 Jeddah, Saudi Arabia; d Department of Inorganic Chemistry, Faculty of Chemistry, University of Tabriz, PO Box 5166616471, Tabriz, Iran

## Abstract

The Pb atom in the polymeric title compound, {[Pb(C_13_H_9_N_3_O_2_)]·CH_3_OH}_*n*_, is five-coordinated within an N_2_O_2_ donor set and a lone pair of electrons, as the *N*-isonicotinamido­salicylaldiminate ligand coordinates the Pb^II^ atom *via* the *O*,*N*,*O*′-donors and simultaneously bridges a neighbouring Pb atom *via* the pyridine N atom; the coordination geometry is based on a trigonal bipyramid with the O atoms in axial positions. The resulting supra­molecular chain is a 3_1_ helix along the *c* axis. These chains are linked *via* inter­molecular Pb⋯O,N inter­actions, as well as O—H⋯O hydrogen bonds.

## Related literature

For crystal engineering studies of metal complexes containing isonicotinylhydrazonate ligands, see: Yuan *et al.* (2007 ▶); Vrdoljak *et al.* (2010 ▶, 2011 ▶). For specialized crystallization techniques, see: Harrowfield *et al.* (1996 ▶).
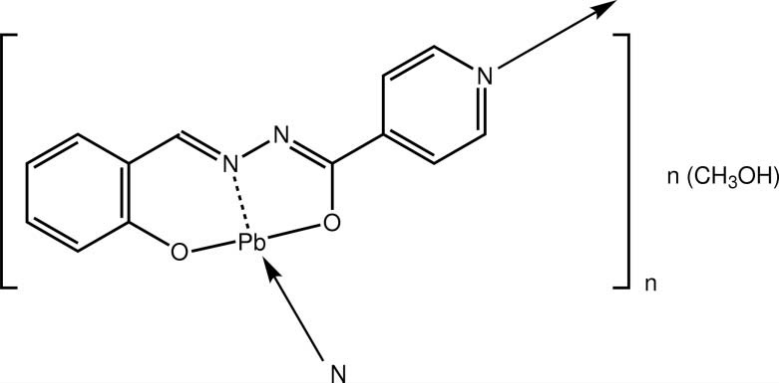


## Experimental

### 

#### Crystal data

[Pb(C_13_H_9_N_3_O_2_)]·CH_4_O
*M* *_r_* = 478.46
Hexagonal, 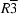

*a* = 28.6702 (5) Å
*c* = 9.0146 (2) Å
*V* = 6417.1 (3) Å^3^
*Z* = 18
Mo *K*α radiation
μ = 11.84 mm^−1^
*T* = 100 K
0.20 × 0.15 × 0.10 mm

#### Data collection

Agilent SuperNova Dual diffractometer with an Atlas detector
Absorption correction: multi-scan (*CrysAlis PRO*; Agilent, 2010 ▶) *T*_min_ = 0.349, *T*_max_ = 1.000
17626 measured reflections
2958 independent reflections
2816 reflections with *I* > 2σ(*I*)
*R*_int_ = 0.026

#### Refinement

*R*[*F* ^2^ > 2σ(*F* ^2^)] = 0.015
*wR*(*F* ^2^) = 0.032
*S* = 1.11
2958 reflections
193 parameters
H-atom parameters constrained
Δρ_max_ = 0.95 e Å^−3^
Δρ_min_ = −0.60 e Å^−3^

### 

Data collection: *CrysAlis PRO* (Agilent, 2010 ▶); cell refinement: *CrysAlis PRO*; data reduction: *CrysAlis PRO*; program(s) used to solve structure: *SHELXS97* (Sheldrick, 2008 ▶); program(s) used to refine structure: *SHELXL97* (Sheldrick, 2008 ▶); molecular graphics: *ORTEP-3* (Farrugia, 1997 ▶) and *DIAMOND* (Brandenburg, 2006 ▶); software used to prepare material for publication: *publCIF* (Westrip, 2010 ▶).

## Supplementary Material

Crystal structure: contains datablock(s) global, I. DOI: 10.1107/S1600536811043078/su2329sup1.cif

Structure factors: contains datablock(s) I. DOI: 10.1107/S1600536811043078/su2329Isup2.hkl

Additional supplementary materials:  crystallographic information; 3D view; checkCIF report

## Figures and Tables

**Table 1:** Hydrogen-bond geometry (Å, °)

*D*—H⋯*A*	*D*—H	H⋯*A*	*D*⋯*A*	*D*—H⋯*A*
O3—H3*o*⋯O1	0.84	1.82	2.659 (3)	172

**Fig. 1.**
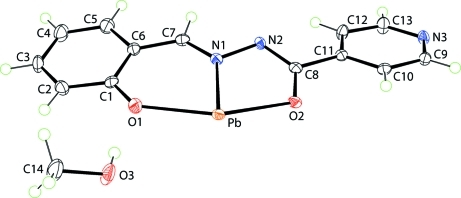
The asymmetric unit of compound (I), showing the atom-labelling scheme and displacement ellipsoids drawn at the 50% probability level.

**Fig. 2.**
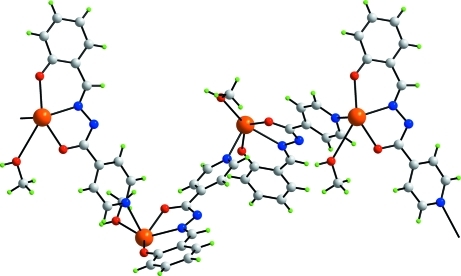
A view of the 31 chain in compound (I), sustained by Pb···N bonds.

**Fig. 3.**
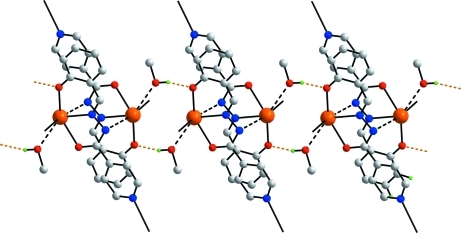
A view of the linear supramolecular chain in compound (I) sustained by Pb···O,N bonds (dashed lines), and O—H···O hydrogen bonds (orange dashed lines).

**Fig. 4.**
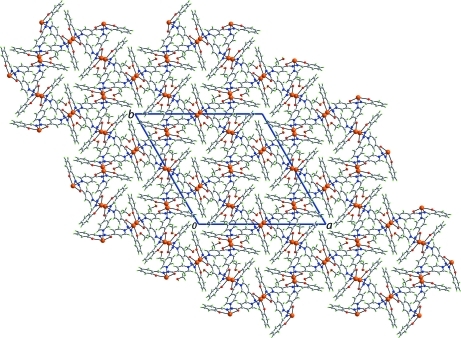
A view in projection down the c axis of the crystal packing in compound (I).

## supplementary crystallographic information

### Comment

2-Oxide-3-benzaldehyde isonicotinylhydrazonato ligands related to that in the title complex, (I), are attracting interest in the context of crystal engineering endeavours (Vrdoljak *et al.*, 2010, 2011). Thus far, the only lead(II) complex reported with this class of ligand is a binuclear complex where the pyridine-N atom does not participate in coordination (Yuan *et al.*, 2007).

The asymmetric unit of compound (I) comprises a Pb^II^ atom, a *N*-isonicotinamidosalicylaldiminato ligand and a methanol molecule of solvation, Fig. 1. The Pb^II^ atom is coordinated by the O,*N*,*O* atoms of the ligand and the pyridine-N atom bridges to a symmetry related Pb^II^ atom. The resultant N_2_O_2_ donor set plus the lone pair of electrons is based on a trigonal bipyramid with the O atoms in axial positions [O1—Pb—O2 = 137.27 (6)°] and the N atoms [N1—Pb—N3^i^ = 90.94 (7)°] and lone pair in equatorial positions; symmetry operation: (i) 1/3 - *x* + *y*, 4/3 - *x*, -2/3 + *z*.

The µ_2_-bridging mode of the tetradentate *N*-isonicotinamidosalicylaldiminato ligand leads to a 3_1_ helical chain along the *c* axis, Fig. 2. The considerable distortions from the ideal geometry arises from the acute chelate angles (O2—Pb—N1 = 66.59 (7)° and O1—Pb—N1 = 73.59 (7)°) as well as the close approach of other donor atoms. Examples of the latter are a methanol-O3 atom [2.959 (3) Å; symmetry operation: 1/3 - *x*, 5/3 - *y*, 5/3 - *z*] and a hydrazine-N2 atom [2.881 (2) Å; symmetry operation: 1/3 - *x*, 5/3 - *y*, 8/3 - *z*]. These interactions along with hydrogen bonding contacts between the methanol molecule of solvation and atom O1 lead to linear chains along the *c* axis (Fig. 3 and Table 1), *i.e*. providing links between the 3_1_ chains mediated by Pb···N interactions, Fig. 4.

### Experimental

A methanol solution (25 ml) of salicylaldehyde (10 mmol) was added drop wise to a methanol solution (15 ml) of 4-pyridinecarboxylic acid hydrazide (10 mmol) and the mixture was stirred for 3 h. The white precipitate was removed by filtration and recrystallized from methanol solution. Then a mixture of the ligand (0.5 mmol) and lead(II) acetate (0.5 mmol) in methanol (35 ml) was stirred at rt for 45 min to give a yellow precipitate which was filtered off and dried. Crystals were obtained by using the branched tube method (Harrowfield *et al.*, 1996). Thus, the complex (0.3 mmol) was placed in the arm to be heated. Methanol was added to fill both arms, and then the arm to be heated was placed in a water bath at 333 K. After 3 days, yellow crystals were deposited in the cooler arm. They were filtered off, washed with water and air dried. Yield: 68%; *M*.pt. 560 K.

### Refinement

C-bound H atoms were placed in calculated positions and were included in the refinement in the riding model approximation: O—H = 0.84 Å, C—H = 0.95 and 0.98 Å, for CH and CH_3_ H atoms, respectively, with *U*_iso_(H) = k × *U*_eq_(O,C), where k = 1.5 for OH and CH_3_H atoms, and k = 1.2 for all other H atoms.

### Crystal data

**Table:**

[Pb(C_13_H_9_N_3_O_2_)]·CH_4_O	*D*_x_ = 2.229 Mg m^−^^3^
*M**_r_* = 478.46	Mo *K*α radiation, λ = 0.71073 Å
Hexagonal, *R*3	Cell parameters from 12065 reflections
Hall symbol: -R 3	θ = 2.4–29.3°
*a* = 28.6702 (5) Å	µ = 11.84 mm^−^^1^
*c* = 9.0146 (2) Å	*T* = 100 K
*V* = 6417.1 (3) Å^3^	Prism, colourless
*Z* = 18	0.20 × 0.15 × 0.10 mm
*F*(000) = 4032	

### Data collection

**Table:**

Agilent SuperNova Dual diffractometer with an Atlas detector	2958 independent reflections
Radiation source: SuperNova (Cu) X-ray Source	2816 reflections with *I* > 2σ(*I*)
Mirror	*R*_int_ = 0.026
Detector resolution: 10.4041 pixels mm^-1^	θ_max_ = 26.5°, θ_min_ = 2.4°
ω scans	*h* = −35→35
Absorption correction: multi-scan (*CrysAlis PRO*; Agilent, 2010)	*k* = −35→35
*T*_min_ = 0.349, *T*_max_ = 1.000	*l* = −11→11
17626 measured reflections	

### Refinement

**Table:**

Refinement on *F*^2^	Primary atom site location: structure-invariant direct methods
Least-squares matrix: full	Secondary atom site location: difference Fourier map
*R*[*F*^2^ > 2σ(*F*^2^)] = 0.015	Hydrogen site location: inferred from neighbouring sites
*wR*(*F*^2^) = 0.032	H-atom parameters constrained
*S* = 1.11	*w* = 1/[σ^2^(*F*_o_^2^) + (0.0114*P*)^2^ + 19.9099*P*] where *P* = (*F*_o_^2^ + 2*F*_c_^2^)/3
2958 reflections	(Δ/σ)_max_ = 0.005
193 parameters	Δρ_max_ = 0.95 e Å^−^^3^
0 restraints	Δρ_min_ = −0.60 e Å^−^^3^

### Special details

**Table:**

Geometry. All e.s.d.'s (except the e.s.d. in the dihedral angle between two l.s. planes) are estimated using the full covariance matrix. The cell e.s.d.'s are taken into account individually in the estimation of e.s.d.'s in distances, angles and torsion angles; correlations between e.s.d.'s in cell parameters are only used when they are defined by crystal symmetry. An approximate (isotropic) treatment of cell e.s.d.'s is used for estimating e.s.d.'s involving l.s. planes.
Refinement. Refinement of *F*^2^ against ALL reflections. The weighted *R*-factor *wR* and goodness of fit *S* are based on *F*^2^, conventional *R*-factors *R* are based on *F*, with *F* set to zero for negative *F*^2^. The threshold expression of *F*^2^ > σ(*F*^2^) is used only for calculating *R*-factors(gt) *etc*. and is not relevant to the choice of reflections for refinement. *R*-factors based on *F*^2^ are statistically about twice as large as those based on *F*, and *R*- factors based on ALL data will be even larger.

### Fractional atomic coordinates and isotropic or equivalent isotropic displacement parameters (Å^2^)

**Table:**

	*x*	*y*	*z*	*U*_iso_*/*U*_eq_	
Pb	0.172066 (4)	0.851864 (4)	1.061072 (10)	0.01156 (4)	
O1	0.09837 (8)	0.86813 (8)	1.0476 (2)	0.0186 (4)	
O2	0.25307 (7)	0.88497 (8)	1.19856 (19)	0.0147 (4)	
O3	0.08109 (8)	0.83045 (11)	0.7716 (2)	0.0318 (6)	
H3o	0.0854	0.8444	0.8561	0.051 (12)*	
N1	0.17051 (9)	0.89277 (9)	1.2955 (2)	0.0119 (4)	
N2	0.21170 (9)	0.90223 (9)	1.3966 (2)	0.0118 (5)	
N3	0.39001 (9)	0.94432 (9)	1.6044 (2)	0.0152 (5)	
C1	0.07467 (11)	0.88735 (11)	1.1315 (3)	0.0144 (6)	
C2	0.03012 (11)	0.89079 (11)	1.0785 (3)	0.0189 (6)	
H2	0.0177	0.8792	0.9803	0.023*	
C3	0.00415 (12)	0.91040 (12)	1.1650 (3)	0.0227 (6)	
H3	−0.0252	0.9129	1.1248	0.027*	
C4	0.02038 (12)	0.92671 (13)	1.3109 (3)	0.0242 (7)	
H4	0.0025	0.9403	1.3702	0.029*	
C5	0.06267 (12)	0.92271 (12)	1.3669 (3)	0.0207 (6)	
H5	0.0733	0.9330	1.4669	0.025*	
C6	0.09089 (11)	0.90384 (11)	1.2809 (3)	0.0145 (6)	
C7	0.13591 (11)	0.90432 (11)	1.3515 (3)	0.0141 (5)	
H7	0.1411	0.9145	1.4531	0.017*	
C8	0.25097 (10)	0.89833 (10)	1.3328 (3)	0.0119 (5)	
C9	0.38059 (11)	0.91341 (11)	1.4841 (3)	0.0146 (5)	
H9	0.4057	0.9022	1.4592	0.017*	
C10	0.33565 (10)	0.89697 (10)	1.3940 (3)	0.0130 (5)	
H10	0.3306	0.8756	1.3084	0.016*	
C11	0.29821 (10)	0.91231 (10)	1.4312 (3)	0.0116 (5)	
C12	0.30750 (11)	0.94353 (11)	1.5581 (3)	0.0170 (6)	
H12	0.2822	0.9539	1.5884	0.020*	
C13	0.35383 (12)	0.95919 (12)	1.6392 (3)	0.0197 (6)	
H13	0.3604	0.9815	1.7235	0.024*	
C14	0.02725 (13)	0.81018 (16)	0.7254 (4)	0.0360 (9)	
H14A	0.0034	0.7974	0.8120	0.054*	
H14B	0.0171	0.7802	0.6562	0.054*	
H14C	0.0239	0.8388	0.6759	0.054*	

### Atomic displacement parameters (Å^2^)

**Table:**

	*U*^11^	*U*^22^	*U*^33^	*U*^12^	*U*^13^	*U*^23^
Pb	0.01132 (6)	0.01153 (6)	0.00909 (5)	0.00365 (4)	−0.00016 (3)	0.00041 (4)
O1	0.0158 (10)	0.0239 (11)	0.0158 (9)	0.0096 (9)	−0.0023 (8)	−0.0010 (8)
O2	0.0146 (10)	0.0187 (10)	0.0091 (9)	0.0071 (8)	−0.0009 (7)	0.0003 (7)
O3	0.0130 (11)	0.0577 (16)	0.0209 (11)	0.0150 (11)	−0.0056 (9)	−0.0155 (11)
N1	0.0111 (11)	0.0087 (11)	0.0128 (10)	0.0027 (9)	−0.0013 (9)	0.0011 (9)
N2	0.0111 (11)	0.0123 (11)	0.0116 (10)	0.0055 (9)	−0.0026 (9)	−0.0009 (9)
N3	0.0129 (12)	0.0180 (12)	0.0151 (11)	0.0081 (10)	−0.0039 (9)	−0.0016 (9)
C1	0.0109 (13)	0.0098 (13)	0.0179 (13)	0.0018 (11)	0.0025 (11)	0.0044 (11)
C2	0.0162 (15)	0.0184 (15)	0.0186 (14)	0.0061 (12)	−0.0030 (11)	0.0027 (12)
C3	0.0137 (15)	0.0267 (17)	0.0297 (16)	0.0117 (13)	−0.0030 (12)	0.0052 (13)
C4	0.0209 (16)	0.0286 (17)	0.0282 (16)	0.0162 (14)	0.0019 (13)	−0.0006 (13)
C5	0.0188 (15)	0.0235 (16)	0.0198 (14)	0.0106 (13)	0.0000 (12)	−0.0007 (12)
C6	0.0116 (13)	0.0110 (13)	0.0180 (13)	0.0035 (11)	0.0003 (11)	0.0029 (11)
C7	0.0160 (14)	0.0129 (13)	0.0113 (12)	0.0057 (12)	0.0009 (11)	−0.0011 (10)
C8	0.0129 (13)	0.0069 (12)	0.0137 (12)	0.0033 (11)	0.0006 (10)	0.0022 (10)
C9	0.0130 (14)	0.0148 (14)	0.0169 (13)	0.0077 (12)	0.0015 (11)	0.0020 (11)
C10	0.0129 (13)	0.0112 (13)	0.0124 (12)	0.0040 (11)	0.0009 (10)	−0.0001 (10)
C11	0.0111 (13)	0.0087 (12)	0.0132 (12)	0.0035 (11)	0.0002 (10)	0.0010 (10)
C12	0.0146 (14)	0.0212 (15)	0.0183 (14)	0.0113 (12)	−0.0012 (11)	−0.0049 (11)
C13	0.0197 (15)	0.0262 (16)	0.0175 (14)	0.0147 (13)	−0.0043 (12)	−0.0086 (12)
C14	0.0178 (16)	0.061 (3)	0.0259 (17)	0.0175 (17)	−0.0050 (13)	−0.0137 (16)

### Geometric parameters (Å, °)

**Table:**

Pb—O1	2.3837 (19)	C3—H3	0.9500
Pb—O2	2.3721 (18)	C4—C5	1.370 (4)
Pb—N1	2.428 (2)	C4—H4	0.9500
Pb—N3^i^	2.530 (2)	C5—C6	1.410 (4)
O1—C1	1.308 (3)	C5—H5	0.9500
O2—C8	1.280 (3)	C6—C7	1.433 (4)
O3—C14	1.413 (4)	C7—H7	0.9500
O3—H3o	0.8400	C8—C11	1.497 (4)
N1—C7	1.295 (3)	C9—C10	1.391 (4)
N1—N2	1.406 (3)	C9—H9	0.9500
N2—C8	1.318 (3)	C10—C11	1.389 (4)
N3—C9	1.340 (3)	C10—H10	0.9500
N3—C13	1.341 (4)	C11—C12	1.393 (4)
N3—Pb^ii^	2.530 (2)	C12—C13	1.380 (4)
C1—C2	1.412 (4)	C12—H12	0.9500
C1—C6	1.426 (4)	C13—H13	0.9500
C2—C3	1.377 (4)	C14—H14A	0.9800
C2—H2	0.9500	C14—H14B	0.9800
C3—C4	1.395 (4)	C14—H14C	0.9800
			
O2—Pb—O1	137.27 (6)	C5—C6—C1	119.5 (3)
O2—Pb—N1	66.59 (7)	C5—C6—C7	116.0 (2)
O1—Pb—N1	73.59 (7)	C1—C6—C7	124.5 (2)
O2—Pb—N3^i^	83.72 (7)	N1—C7—C6	128.7 (2)
O1—Pb—N3^i^	82.01 (7)	N1—C7—H7	115.6
N1—Pb—N3^i^	90.94 (7)	C6—C7—H7	115.6
C1—O1—Pb	138.69 (17)	O2—C8—N2	127.5 (2)
C8—O2—Pb	115.50 (16)	O2—C8—C11	118.0 (2)
C14—O3—H3o	109.5	N2—C8—C11	114.6 (2)
C7—N1—N2	112.0 (2)	N3—C9—C10	122.9 (3)
C7—N1—Pb	131.62 (18)	N3—C9—H9	118.6
N2—N1—Pb	116.13 (15)	C10—C9—H9	118.6
C8—N2—N1	111.8 (2)	C11—C10—C9	118.9 (2)
C9—N3—C13	117.9 (2)	C11—C10—H10	120.6
C9—N3—Pb^ii^	119.49 (17)	C9—C10—H10	120.6
C13—N3—Pb^ii^	121.59 (18)	C10—C11—C12	118.2 (2)
O1—C1—C2	120.8 (2)	C10—C11—C8	120.7 (2)
O1—C1—C6	122.2 (2)	C12—C11—C8	121.0 (2)
C2—C1—C6	116.9 (2)	C13—C12—C11	119.2 (3)
C3—C2—C1	122.0 (3)	C13—C12—H12	120.4
C3—C2—H2	119.0	C11—C12—H12	120.4
C1—C2—H2	119.0	N3—C13—C12	123.0 (3)
C2—C3—C4	120.8 (3)	N3—C13—H13	118.5
C2—C3—H3	119.6	C12—C13—H13	118.5
C4—C3—H3	119.6	O3—C14—H14A	109.5
C5—C4—C3	118.7 (3)	O3—C14—H14B	109.5
C5—C4—H4	120.6	H14A—C14—H14B	109.5
C3—C4—H4	120.6	O3—C14—H14C	109.5
C4—C5—C6	122.1 (3)	H14A—C14—H14C	109.5
C4—C5—H5	119.0	H14B—C14—H14C	109.5
C6—C5—H5	119.0		
			
O2—Pb—O1—C1	−23.4 (3)	O1—C1—C6—C7	3.9 (4)
N1—Pb—O1—C1	−1.6 (3)	C2—C1—C6—C7	−178.4 (3)
N3^i^—Pb—O1—C1	−95.0 (3)	N2—N1—C7—C6	175.1 (3)
O1—Pb—O2—C8	34.3 (2)	Pb—N1—C7—C6	−10.7 (4)
N1—Pb—O2—C8	11.55 (17)	C5—C6—C7—N1	−174.9 (3)
N3^i^—Pb—O2—C8	105.32 (18)	C1—C6—C7—N1	3.1 (5)
O2—Pb—N1—C7	172.3 (3)	Pb—O2—C8—N2	−9.3 (3)
O1—Pb—N1—C7	8.1 (2)	Pb—O2—C8—C11	171.80 (17)
N3^i^—Pb—N1—C7	89.5 (2)	N1—N2—C8—O2	−3.6 (4)
O2—Pb—N1—N2	−13.71 (15)	N1—N2—C8—C11	175.3 (2)
O1—Pb—N1—N2	−177.83 (18)	C13—N3—C9—C10	0.9 (4)
N3^i^—Pb—N1—N2	−96.45 (17)	Pb^ii^—N3—C9—C10	−167.6 (2)
C7—N1—N2—C8	−170.4 (2)	N3—C9—C10—C11	−1.2 (4)
Pb—N1—N2—C8	14.4 (3)	C9—C10—C11—C12	−0.1 (4)
Pb—O1—C1—C2	179.32 (19)	C9—C10—C11—C8	176.4 (2)
Pb—O1—C1—C6	−3.1 (4)	O2—C8—C11—C10	−17.9 (4)
O1—C1—C2—C3	179.4 (3)	N2—C8—C11—C10	163.1 (2)
C6—C1—C2—C3	1.7 (4)	O2—C8—C11—C12	158.5 (2)
C1—C2—C3—C4	−1.4 (5)	N2—C8—C11—C12	−20.5 (4)
C2—C3—C4—C5	−0.2 (5)	C10—C11—C12—C13	1.7 (4)
C3—C4—C5—C6	1.5 (5)	C8—C11—C12—C13	−174.8 (3)
C4—C5—C6—C1	−1.2 (4)	C9—N3—C13—C12	0.9 (4)
C4—C5—C6—C7	177.0 (3)	Pb^ii^—N3—C13—C12	169.1 (2)
O1—C1—C6—C5	−178.1 (3)	C11—C12—C13—N3	−2.2 (5)
C2—C1—C6—C5	−0.4 (4)		

Symmetry codes: (i) −*x*+*y*−1/3, −*x*+4/3, *z*−2/3; (ii) −*y*+4/3, *x*−*y*+5/3, *z*+2/3.

### Hydrogen-bond geometry (Å, °)

**Table:**

*D*—H···*A*	*D*—H	H···*A*	*D*···*A*	*D*—H···*A*
O3—H3o···O1	0.84	1.82	2.659 (3)	172

## References

[bb1] Agilent (2010). *CrysAlis PRO* Agilent Technologies, Yarnton, Oxfordshire, England.

[bb2] Brandenburg, K. (2006). *DIAMOND* Crystal Impact GbR, Bonn, Germany.

[bb3] Farrugia, L. J. (1997). *J. Appl. Cryst.* **30**, 565.

[bb4] Harrowfield, J. M., Miyamae, H., Skelton, B. W., Soudi, A. A. & White, A. H. (1996). *Aust. J. Chem.* **49**, 1165–1169.

[bb5] Sheldrick, G. M. (2008). *Acta Cryst.* A**64**, 112–122. 10.1107/S0108767307043930 18156677

[bb6] Vrdoljak, V., Prugovečki, B., Matković-Čalogović, D. R., Dreos, R., Siega, P. & Tavagnacco, C. (2010). *Cryst. Growth Des.* **10**, 1373–1382.

[bb7] Vrdoljak, V., Prugovečki, B., Matković-Calogović, D. & Pisk, P. (2011). *CrystEngComm*, **13**, 4382–4390.

[bb8] Westrip, S. P. (2010). *J. Appl. Cryst.* **43**, 920–925.

[bb9] Yuan, Y.-Z., Zhou, J., Liu, X., Liu, L.-H. & Yu, K.-B. (2007). *Inorg. Chem. Commun.* **10**, 475–478.

